# The WHO's Global Action Plan on Dementia

**DOI:** 10.1002/alz70860_110731

**Published:** 2025-12-23

**Authors:** Katrin M. Seeher

**Affiliations:** ^1^ World Health Organization, Geneva, Switzerland

## Abstract

The Global Action Plan helps guide countries in addressing the dementia public health crisis. For several reasons, including COVID‐19, action has been slowed in many countries, resulting in the World Health Assembly recently extending the Plan to 2031. This presentation will review what the Plan is and the prospects for implementation by the end of the decade.

